# Changes in feeding habits promoted the differentiation of the composition and function of gut microbiotas between domestic dogs (*Canis lupus familiaris*) and gray wolves (*Canis lupus*)

**DOI:** 10.1186/s13568-018-0652-x

**Published:** 2018-08-02

**Authors:** Tianshu Lyu, Guangshuai Liu, Huanxin Zhang, Lidong Wang, Shengyang Zhou, Huashan Dou, Bo Pang, Weilai Sha, Honghai Zhang

**Affiliations:** 1 Qufu Normal University, Qufu, 273165 China; 2 Ocean University of China, Tsingtao, 266100 China; 3 Dalai Lake National Nature Reserve Management Bureau, Hailar, 021000 China

**Keywords:** Metagenomic, Gut microbiota, *Canis lupus*, *Canis lupus familiaris*, Function comparison

## Abstract

Wolves (*Canis lupus*) and their domesticated and close relatives, dogs (*Canis lupus familiaris*), have great differences in their diets and living environments. To the best of our knowledge, the fundamental question of how the abundance and function of the gut microbiota of domestic dogs evolved to adapt to the changes in host feeding habits has yet to be addressed. In this study, our comparative analyses of gut metagenomes showed that the abundance of gut microbiota between the two species have some significant differences. Furthermore, a number of taxa observed in higher numbers in domestic dogs are related to carbohydrate metabolism, which may be because that there were more complicated polysaccharides in dogs diets than that in wolves diets. A significant difference in the abundance of genes encoding glycosyltransferase family 34 (GT34), carbohydrate-binding module family 25 (CBM25), and glycoside hydrolase family 13 (GH13) between the gut microbiota metagenomes of domestic dogs and gray wolves also supported this observation. Furthermore, the domestic dog gut microbiota has greater valine, leucine and isoleucine biosynthesis and nitrogen metabolism. This result showed that compared with wolves, the domestic dog diet contains a smaller amount of animal protein, which is consistent with the dietary composition of wolves and dogs. Our results indicate that the function and abundance of gut microbiota of domestic dogs has been adapted to domestication, which is of great significance for the ability of domestic dogs to adapt to changes in food composition.

## Introduction

The domestication of animals and plants has had an important impact on the progress of human civilization (Diamond 2002). The discovery and domestication of plants and animals transformed human civilization from the primitive hunting lifestyle to the traditional pattern of agricultural farming, greatly promoting the development of human civilization (Zeder 2015; Zohary and Hopf 2000). The selective breeding of animals and plants during the process of domestication has significantly altered their genes and traits compared to those of wild populations (Bruford et al. 2003; Giuffra et al. 2000; Rubin et al. 2010). Canines are carnivores whose wide distribution is closely related to humans. Some studies have shown that dogs, the first domesticated vertebrate, were domesticated by humans from wolves in East Asia approximately 15,000 years ago (Savolainen et al. 2002). However, recent findings suggest that the domestication of dogs may have occurred earlier, during the late Pleistocene in modern day Belgium (Germonpré et al. 2009), the Czech Republic (Germonpré et al. 2012), and southwestern Siberia (Ovodov et al. 2011). Therefore, the timing and location of dog domestication is still unclear (Larson et al. 2012; Perri 2016). In dogs, genes for digestion, metabolism, nerves and tumors show a coevolutionary trend with humans (Wang et al. 2013), and the increased ability of dogs to digest starch in food relative to wolves has been demonstrated at the genome level (Axelsson et al. 2013). A mitochondrial genomes study for present-day dogs and wolves and fossil canids suggested that wolves may come into contact with European hunters and share the food from hunters, thus, Europeans domesticated wolves and turned them into dogs (Thalmann et al. 2013). Bosch et al. (2015) suggested that as real carnivores, the main food source of wild wolves were mammals including ungulates and non-ungulates. Although wild wolves sometimes choose to eat some vegetations, the amount of vegetal matters they eat is negligible compared to the amount of meat they eat. Gao et al. (1996) and Yan et al. (2006) found that in China, the main food sources of wild wolves lived in Inner Mongolia were ungulates, including a considerable number of livestock. Unlike wild wolves, present-day dogs have a more diverse diet, mainly including starch food, fat and protein (Lasater and Mooers 1993; Rowe et al. 1997). However, although the whole genome characteristics of wolves and domestic dogs has been well studied, the differences in the structure and function of digestive tract flora between wolves and domestic dogs remains open to question.

There are 10^13^–10^14^ gut microbiota living in the human digestive tract, which are closely associated with host digestive physiology, and these microbes form a large and complex ecosystem (Eckburg et al. 2005; Gill et al. 2006). The gut microbiota is important to mammals, affecting many aspects of animal physiology and health, such as digestion and nutrient absorption, energy supply, fat metabolism, and immune regulation (Guarner and Malagelada 2003). A comprehensive understanding of the composition and structure of the intestinal microbiota is the first step to reveal the interactions between intestinal microorganisms and animal hosts. In 2015, a study on the gut microbiota of Hadza hunter-gatherers indicated that the complex polysaccharides in the diet of this group made their gut microbiota adapted to metabolize a broad range of carbohydrates (Rampelli et al. 2015). Another study also confirmed that the variety of polysaccharides in food has an effect on the composition and function of gut microbiota (Hehemann et al. 2010). In addition, there are a number of phenomena of gut microbiota and host adaptive evolution in wild animals. A previous study revealed that the gut microbiota of the giant panda helps the host to digest cellulose, since the giant panda lacks the gene encoding cellulase (Zhu et al. 2011). Although creosote toxins in plants may be harmful to many herbivores, the function and structure of gut microbiota of the desert woodrat has changed to help the host adapt to the toxins in such plants and allow it to use such plants as a food source (Kohl et al. 2014).

Metagenomics was the first proposed method to study the whole genome information contained in a microbial community (Handelsman et al. 1998) and was defined as a genomics technology to study the microbial community without culturing individual microorganisms (Chen and Pachter 2005). In this study, we sequenced metagenomic DNA using an Illumina HiSeq platform (Novo gene) to identify the genes and genomes from the intestinal microbiota in feces from dogs and wolves. The main goal of this study was to compare the composition and function of gut microbiotas between dogs and wolves and screen the gut microbiota and functions of dogs that are significant different from wolves, which is meaningful for understanding that domestic dogs can eat a much more complex variety of foods compared to wolves.

## Materials and methods

### Sample collection

All fecal samples were collected from four adult wolves and three adult dogs living in the Dalai Lake National Nature Reserve in the Inner Mongolia region of northern China during November and December 2016. These samples were divided into two groups: the four wolf fecal samples were named CL1.1–CL1.4, and the three dog fecal samples were named CL2.1–CL2.3 (Table 1). The wolves were enclosure in iron fences and have plenty of room to move. The diets of the four wolves included raw chicken, sheepskin, and lamb to simulate the food composition of wild wolves as much as possible, and the diets of the three dogs included leftover foods, including vegetables, steamed buns, noodles, fruits and meats. The selected fecal samples were collected from the surface of fresh snow after no more than 2 h to ensure the samples were clean and unmistakable. The fecal samples were collected at an ambient temperature of − 20 °C and were stored at − 80 °C in an Ultra-Low Temperature Freezer before DNA extraction to guarantee the integrity of the DNA from intestinal flora.

**Table 1:** Sample information

Sample name	Sex	Age	Local
CL1.1	Male	7	Dalai Lake National Nature Reserve
CL1.2	Female	7	Dalai Lake National Nature Reserve
CL1.3	Male	4	Dalai Lake National Nature Reserve
CL1.4	Female	4	Dalai Lake National Nature Reserve
CL2.1	Female	6	Dalai Lake National Nature Reserve
CL2.2	Male	4	Dalai Lake National Nature Reserve
CL2.3	Male	4	Dalai Lake National Nature Reserve

### DNA extraction

Genomic DNA was extracted from feces using a phenol–chloroform extraction method following the guidelines for subsequent analysis (Köchl et al. 2005). The potential contamination and degree of degradation of the extracted DNA was monitored by 1% AGE (agarose gel electrophoresis). Next, we used a NanoPhotometer^®^ spectrophotometer (IMPLEN, CA, USA) to assess the purity of the DNA, and a Qubit^®^ dsDNA Assay kit was used to measure the concentration of DNA with a Qubit^®^ 2.0 fluorometer (Life Technologies, CA, USA). Only DNA samples with concentrations above 1 µg were used for subsequent library construction.

### Metagenomic sequencing

The qualifying DNA samples were broken into approximately 350 bp fragments using ultrasonic waves (Focused-ultrasonication with AFA Technology, Covaris, UK). The fragments were end-polished, A-tailed, purified, ligated with full-length adaptors and further PCR amplificated to generate libraries. After construction, the libraries were diluted to 2 ng/µl using a Qubit 2.0 (Invitrogen, USA). Next, the fragment sizes in the library were determined using an Agilent 2100 Bioanalyzer (Agilent, USA), and the effective concentration (> 3 nM) of the library was accurately quantified using Real-time Quantitative PCR Detecting System (Real-time q-PCR) (Panaro et al. 2000; Smith and Osborn 2009). The index-coded sample clustering was conducted with a cBot cluster generation system following the manufacturer’s instructions. An Illumina HiSeq xten platform (Illumina, USA) was used to sequence the libraries and generated paired-end reads after cluster generation. The data set supporting the results of this article is available in the Sequencing Read Archive (SRA) database, accession numbers SRP179020.

### Data analysis

After sequencing, more than 6.4 Gb of sequences was generated for each DNA sample. The Short Oligonucleotide Analysis Package aligner (SOAP aligner) was used to remove reads with low quality scores, large numbers of “N” bases, or those contaminated with adapters to obtain clean reads (Gu et al. 2013). The effective rates of clean data of samples are greater than 99.4%. Short Oligonucleotide Analysis Package denovo (SOAP denovo) was used to finish the assembly analysis of clean data (Luo et al. 2012). The length distribution of scarftigs were acceptable for subsequent analysis after treatment by SOAP denovo. The open reading frame (ORF) prediction of scarftigs and subsequent results (gene catalogue) were performed using MetaGeneMark and CD-HIT (Fu et al. 2012; Karlsson et al. 2013; Li et al. 2014; Mende et al. 2012; Oh et al. 2014). Basic statistics were performed based on the abundances of various genes in the samples in the gene catalogue (Table 2).

**Table 2:** Basic information of the gene catalog

Basic information	
ORFs NO.	307,207
Integrity:end^a^	56,321 (18.33%)
Integrity:start	67,985 (22.13%)
Integrity:all^b^	159,718(51.99%)
Integrity:none	23,183 (7.55%)
Total Len. (Mbp)^c^	218.5
Average Len. (bp)^d^	711.26
GC percent^e^	42.29

^a^Genes that only contain termination/initiation codons

^b^Genes that contain none/all codons

^c^Overall length of genes

^d^Average length of genes

^e^Estimate of the total GC content of genes

We compared unigenes with the sequences of bacteria, fungi, archaea and viruses, which were extracted from NR database of NCBI (Version: 2016-11-05) using DIAMOND (Buchfink et al. 2015). Correlation analysis to assess correlations between samples, clustering analysis to assess the similarities of bacterial taxa between samples at the phylum and genus levels, and principal component analysis (PCA) and non-metric multidimensional scaling (NMDS) to assess the significantly different bacterial taxa and gene functions between species were all calculated with R software. ANOSIM analysis was used to test whether the differences between groups were significantly greater than those within the group to assess the importance of the grouping (Clarke 1993), which was calculated using QIIME (Version: 1.7.0). Metastats was used to identify the species with significant differences (White et al. 2009). In addition, DIAMOND was used to compare the unigenes with the Kyoto Encyclopedia of Genes and Genomes (KEGG) and Carbohydrate-Active Enzymes Database, Version: 2014.11.25 (CAZy) databases to calculate the relative abundances of the gut microbiota at different functional levels. The basic steps of functional annotations are as follows: (1) DIAMOND software was used to compare unigenes with various functional databases (BLASTp, e value is ≤ 1e−5) (Feng et al. 2015); (2) screening of comparisons of results and the results of the highest score comparison (one HSP > 60 bits) were selected for subsequent analysis (Li et al. 2014); (3) relative abundances at different functional levels were compared based on the results of the comparison, where the relative abundance of each functional level is equal to the sum of the relative abundance of the genes in the function hierarchy (Karlsson et al. 2013); (4) the number of genes in each sample for each classification level table were based on the results of the functional annotation and gene abundance tables; and (5) the dimension reduction analysis and sample cluster analysis of Bray–Curtis distances were based on the abundance of functions.

## Results

A total of 43 Gb high-quality sequences were obtained from the samples of seven individual animals. After quality control and filtering, the total amount of clean data also remained at about 43 Gb, with an effective percentage of more than 99.4% (Table 3).

**Table 3:** The statistical table of data

Sample	Insert size (bp)	Raw data	Clean data	GC %	Effective %
CL1.1	350	6407.94	6403.51	43.83	99.931
CL1.2	350	6636.36	6628.6	42.97	99.883
CL1.3	350	6787.35	6771.63	38.61	99.768
CL1.4	350	6210.43	6201.88	43.65	99.862
CL2.1	350	6327.63	6323.43	42.04	99.934
CL2.2	350	6459.95	6449.21	44.28	99.834
CL2.3	350	6229.68	6192.29	42.39	99.4

### Correlation analysis of fecal microbiomes

**Fig. 1:**
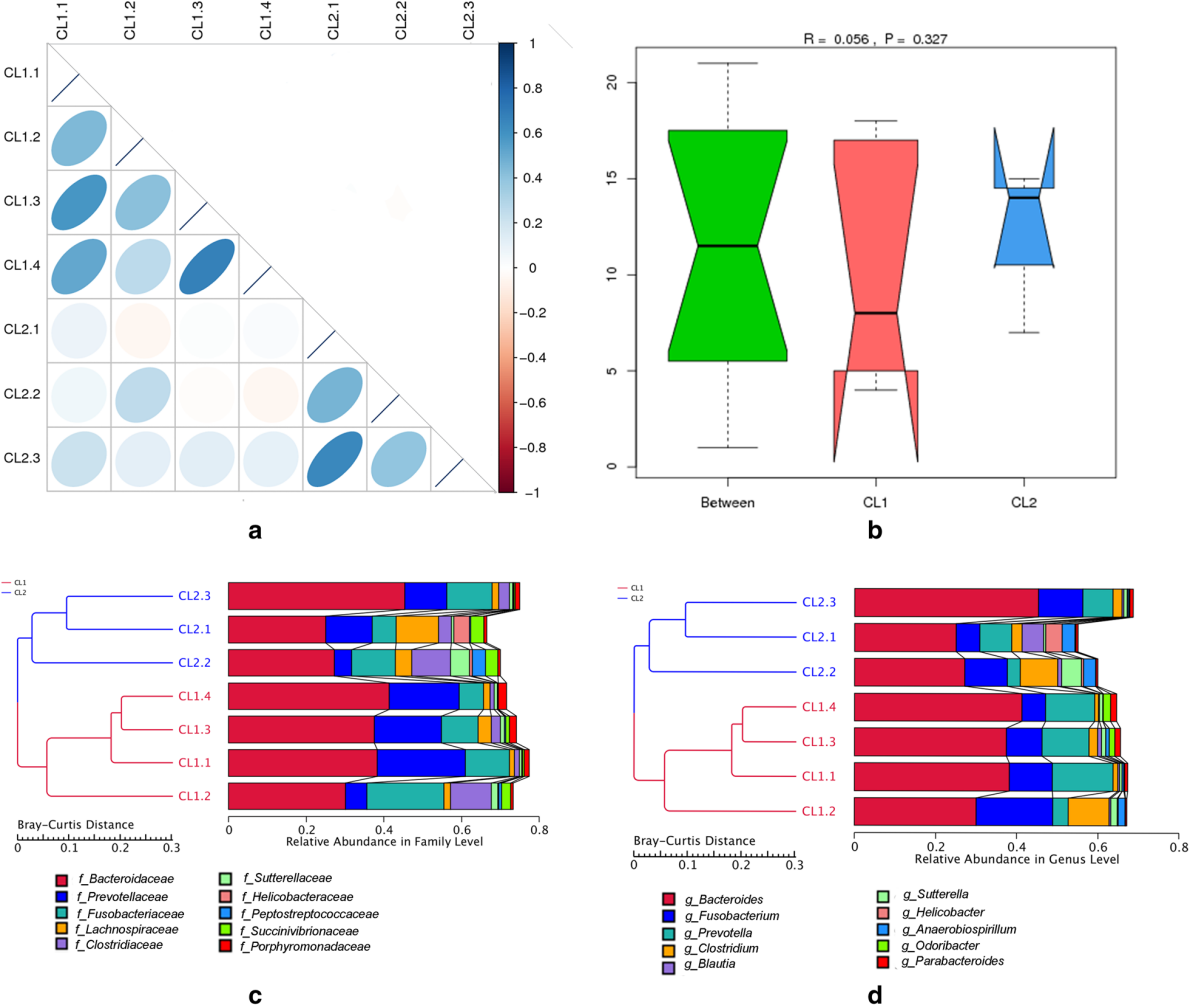
Correlation coefficient between samples, the ANOSIM analysis based on species, and the cluster analysis based on Bray–Curtis distance. **a** Correlation coefficient. The different colors represent the different Spearman correlation coefficient, the deeper the color is, the greater the absolute value of the correlation between samples; **b** ANOSIM analysis at the phylum level. The x-axis for grouping information, y-axis for distance information, R-value is between (− 1, 1) and R-value is greater than 0, indicating there are significant differences between groups; **c** the cluster analysis at the family level; and **d** the cluster analysis at the genus level

The result of the heatmap of correlation coefficients revealed that the correlation within groups is stronger than that between groups (Fig. 1). The ANOSIM analysis based on species richness and the cluster analysis based on Bray–Curtis distance reach the same conclusion (Fig. 1). These results indicated that the grouping is meaningful and there was a difference in bacterial community structure between the two species.

### Comparison of significantly different microbial groups

**Fig. 2:**
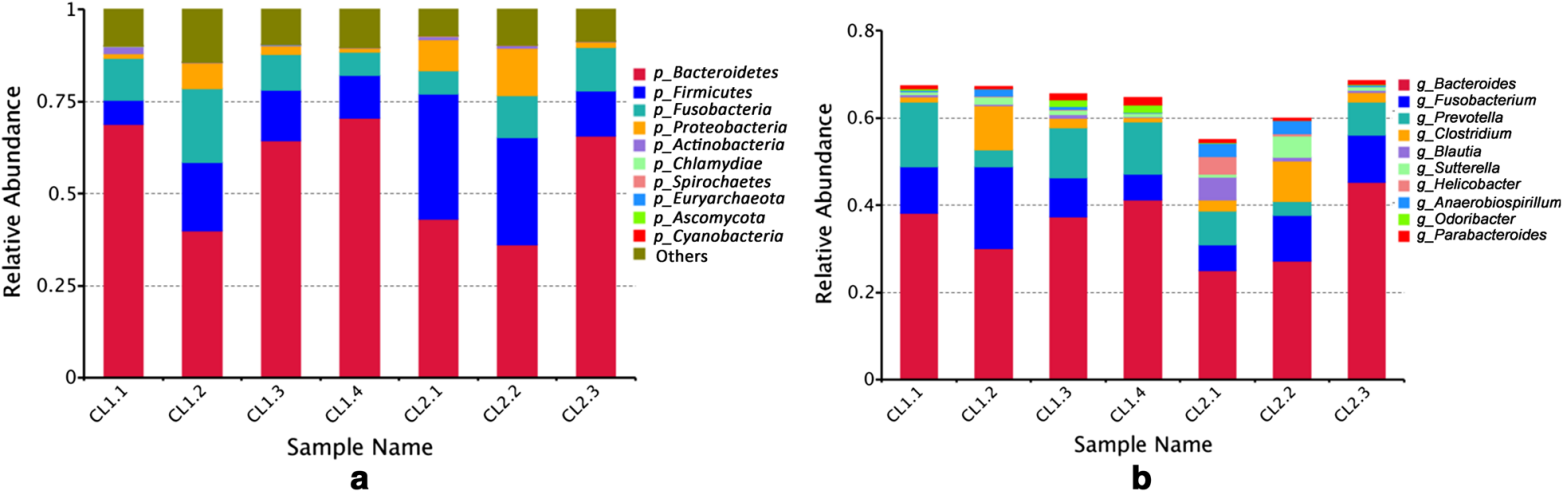
The relative abundances of dominant bacteria in each sample at **a** the phylum level and **b** the genus level

The top 10 taxa with the maximum relative abundances were selected for each sample (Fig. 2). The results revealed that the top five phyla present in the microbiomes both of wolves and dogs were as follows: *Bacteroidetes*, *Fusobacteria*, *Firmicutes*, *Proteobacteria*, and *Actinobacteria*. The top five genera in the microbiomes both of wolves and dogs were *Bacteroides*, *Fusobacterium*, *Prevotella*, *Clostridium*, and *Blautia*.

**Fig. 3:**
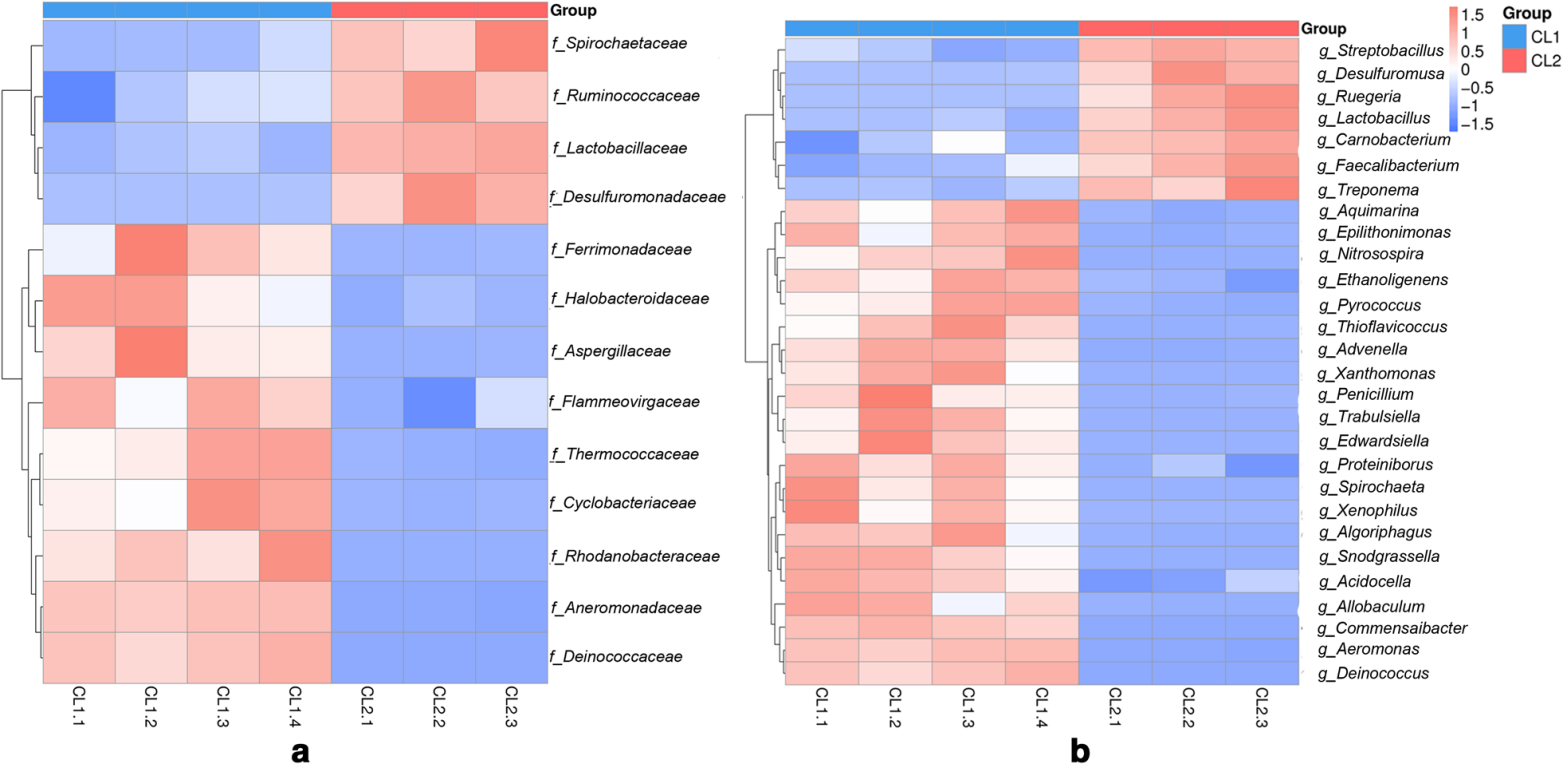
The abundance clustering based on significant differences in species at **a** the family level and **b** the genus level. Horizontal represents sample information; vertical information represents annotated information of species; the cluster tree on the left is the species cluster tree. The values corresponding to the intermediate heat map are the Z values of the relative abundance of each row of species after standardized treatment

Metastat was used to study the significant differences in species abundance between the two groups at different taxonomic levels. The species with significant differences were selected according to the q-values, and the abundance distributions of the different species between the groups are shown in (Fig. 3). At the family level, the bacterial taxa with significantly higher abundances (q < 0.05) in dogs than in wolves were *Ruminococcaceae*, *Spirochaetaceae*, *Lactobacillaceae*, and *Desulfuromonadaceae*. The difference of *Lactobacillaceae* was observed to be extremely significant in dogs and wolves (q < 0.01). At the genus level, the bacterial taxa that had significantly higher abundances in dogs than that in wolves were *Streptobacillus*, *Desulfuromusa*, *Ruegeria*, *Lactobacillus*, *Carnobacterium*, *Faecalibacterium* and *Treponema*.

### Comparison of significantly different functions

**Fig. 4:**
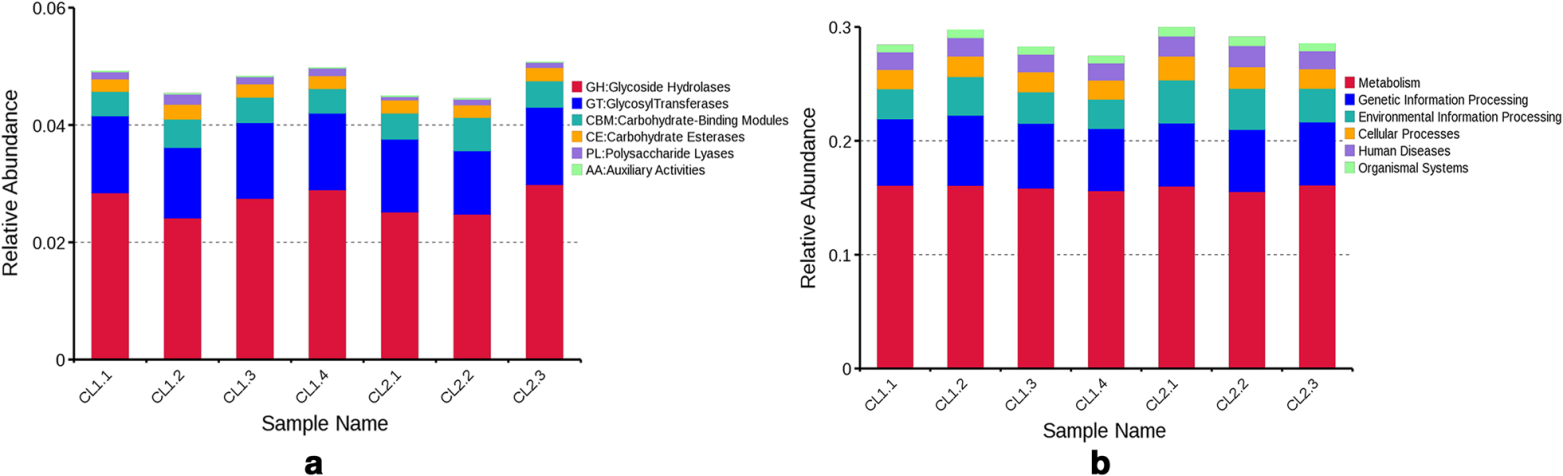
The relative abundance of functionally annotated genes at level 1 **a** based on the CAZy database and **b** based on the KEGG database. The vertical axis represents the relative proportion of comments to a functional class. The horizontal axis represents the sample name. The functional categories corresponding to each color block are shown in the figure on the right

**Fig. 5:**
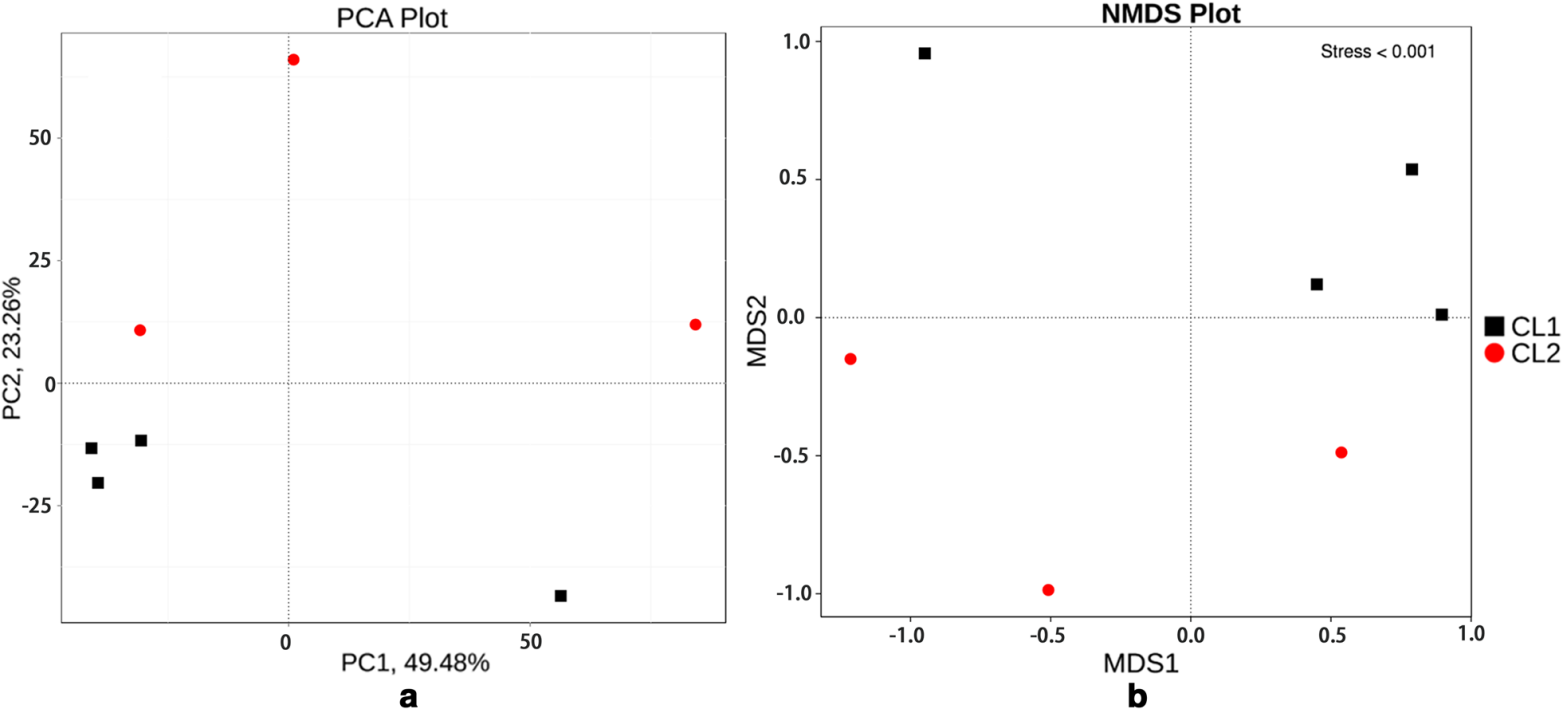
The PCA and NMDS dimension reduction analysis based on the functional gene abundance using KEGG databases. **a** PCA analysis; **b** NMDS dimension reduction analysis

KEGG database and CAZy database were selected separately in order to conduct metabolic pathway enrichment and enzymatic differentiation analysis on gut microbiota of wolves and dogs. After analyzing the metagenomes using two databases, we observed that the top three functional categories at the second CAZy classification level were glycoside hydrolases (GHs, CL1, 13.1 ± 2.8; CL2, 15.8 ± 2.0), glycosyltransferases (GTs, CL1, 12.6 ± 1.6; CL2, 14.7 ± 1.2), and carbohydrate-binding modules (CBMs, CL1, 13.9 ± 3; CL2, 14.7 ± 1.6). In the KEGG database, the top three dominant functional gene categories were metabolism (CL1, 13.6 ± 2.5; CL2, 15.1 ± 1.5), genetic information (CL1, 14.0 ± 2.7; CL2, 14.6 ± 1.5), and environmental information processing (CL1, 13.2 ± 3.1; CL2, 15.5 ± 2.1) (Fig. 4). Within the metabolism classification, carbohydrate metabolism (CL1, 13.5 ± 2.5; CL2, 15.3 ± 1.6), global and overview maps (CL1, 13.2 ± 2.7; CL2, 15.6 ± 1.8), and amino acid metabolism (CL1, 13.3 ± 2.6; CL2, 15.5 ± 1.9) were the most represented gene categories. At the third level, the most represented gene categories were for amino acid biosynthesis (CL1, 13.1 ± 2.8; CL2, 15.8 ± 2.0), which was within the global and overview maps. The PCA and NMDS analysis based on the annotation of two databases revealed that there were a number of significant differences with respect to microbial functions between wolves and dogs (Fig. 5).

According to the annotated KEGG results, no significant differences in functional categories were observed between dogs and wolves at the first and second levels, but differences were observed at the third level (p < 0.05). The functional categories with significantly higher abundances in domestic dogs than wolves at the third level were valine, leucine and isoleucine biosynthesis (ko00290) and nitrogen metabolism (ko00910). We also observed that the domestic dog metagenome has a higher enrichment for GT34, CBM25, and GH13 (p < 0.01) compared to wolves. It is worth noting that we did not observe carbohydrate-binding module 25 in wolves. At the third level, six enzymes belonging to the glycosyltransferase and five enzymes belonging to the glycoside hydrolase showed significantly higher abundances in domestic dogs compared to wolves (q < 0.05). In addition, we observed that seven of these enzymes act on a starch and sucrose metabolic pathway (ko00500), including 4-alpha-glucanotransferase (EC:2.4.1.25), glycogen branching enzyme (EC:2.4.1.18), alpha-1,4-glucan: phosphate-alpha-maltosyltransferase (EC:2.4.99.16), amylosucrase (EC:2.4.1.4), cyclomaltodextrin glucanotransferase (EC:2.4.1.19), alpha-amylase (EC:3.2.1.1), and cyclomaltodextrinase (EC:3.2.1.54) (Fig. 7).

## Discussion

By comparing the gut microbiota of wolves and domestic dogs, we observed that the differences in microbial species and genes were related to many functions, such as starch and cellulose metabolism. The top five most abundant phyla in domestic dogs and wolves were *Bacteroidetes*, *Fusobacteria*, *Firmicutes*, *Proteobacteria*, and *Actinobacteria*, similar to the results of (Wu et al. 2017). At other taxonomic levels, such as family and genus, no significant differences were observed in the top five taxa between dogs and wolves (q < 0.05). The microbial taxa with significantly higher abundances in domestic dogs significantly correlated with their complex polysaccharide diet, including the families *Ruminococcaceae* (Brown and Brown 1966; Huws et al. 2011) and *Desulfuromonadaceae* (Greene 2014), which are related to cellulose digestion, and *Lactobacillaceae*, members of which are related to the fermentation of glucose, as well as the genera *Streptobacillus* (Gharbia and Edwards 2015), *Desulfuromusa* (Werner and Kai 2015), *Lactobacillus* (Zaunmüller et al. 2006), *Carnobacterium* (Leisner et al. 2007), and *Faecalibacterium* (Lopezsiles et al. 2012). These results indicate that the composition and function of gut microbiota in domesticated dogs may have been influenced by human food.

Although PCA and NMDS analyses based on the comments of carbohydrate-active enzymes and metabolic pathway were able to divide dogs and wolves into two populations, the annotation results for the top 6 functions of gut microbiota based for the two databases showed no significant differences (Fig. 4). Therefore, we used a metastat analysis to evaluate all the functions of the annotations, including non-essential functions. According to the metastat analysis, the significant differences in bacterial functions were identified based on both of the databases.

**Fig. 6:**
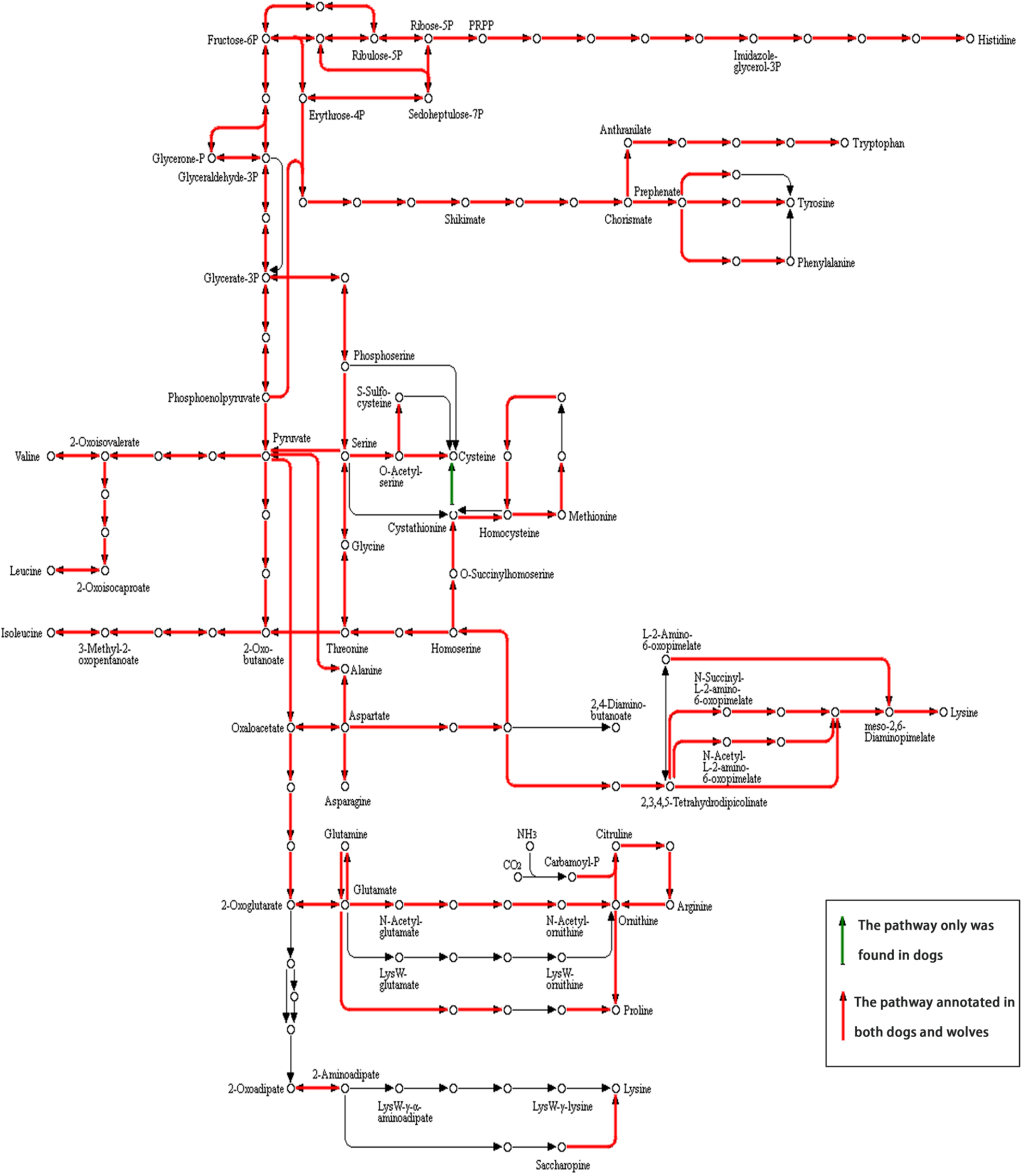
The annotated pathway of amino acids biosynthesis

The KEGG database analysis showed that the gut microbiota of domestic dogs was characterized by an enrichment of genes involved in carbohydrate metabolism (CL1, 13.5 ± 2.5; CL2, 15.3 ± 1.6), global and overview maps (CL1, 13.2 ± 2.7; CL2, 15.6 ± 1.8), and amino acid metabolism (CL1, 13.3 ± 2.6; CL2, 15.5 ± 1.9). The higher gene percentages in these classifications suggested a complex diet intake and an ability for polysaccharide absorption in domestic dogs. In particular, the significantly higher abundance of genes involved in valine, leucine and isoleucine biosynthesis (ko00290) and nitrogen metabolism (ko00910) in domestic dogs also revealed that the gut microbiota of domestic dogs is more active in branched-chain amino acid (BCAA) metabolism. Some previous studies of gut microbiota also showed that the significant differences in these two pathways corresponded to a low animal protein intake (Rampelli et al. 2015; Schnorr et al. 2014). This result is in line with the low contribution of meat to the diets of domestic dogs. In addition, we observed that the annotated pathway Biosynthesis of amino acids (ID: map01230) was significantly different between wolves and domestic dogs (Fig. 6). In this, metabolic pathway l-cysteine is produced from l-cystathionine by cystathionine gamma-lyase (EC:4.4.1.1). This amino acid is widely present in most high-protein foods, including animal and plant sources. Cysteine is found in many meat products (including pork, sausage meat, chicken, turkey, duck and lunch meat), eggs and dairy. In plant-based foods, red peppers, garlic, onions, broccoli, brussels sprout, oats, granola, wheat germ and sprouted lentils have a high cysteine content. Cysteine plays an important role in animals, and the lack of this amino acid can lead to several diseases (Ames 1999; Chévezbarrios et al. 2000; Goodman et al. 2000; Novelli et al. 2009; Silva et al. 2013). The gut microbiota of domestic dogs can synthesize cysteine more efficiently than wolves by producing cysteine using cystathionine gamma-lyase directly. We speculated that wolves ingest sufficient amounts of cysteine because the foods wolves eat are rich in cysteine. The diet of dogs has more starch and cellulose than cysteine. To avoid diseases caused by a lack of cysteine, the gut microbiota needs to supply a large amount of cysteine to their hosts. Thus, they possess a more efficient way of synthesizing cysteine. This result may be due to the reduction of cysteine intake by dogs from food.

**Fig. 7:**
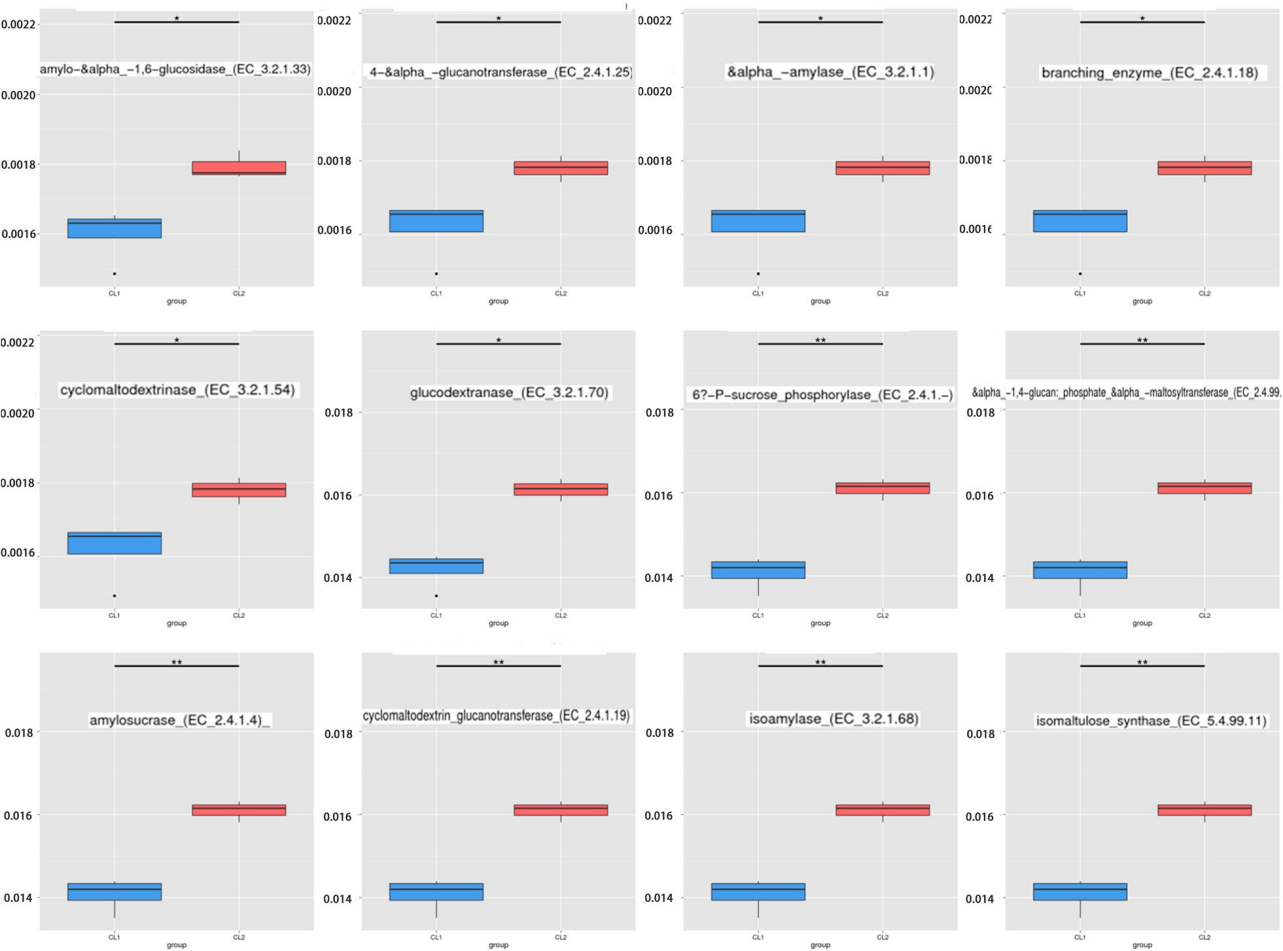
Significantly different enzymes based on the CAZy database

According to the analysis using the CAZy second level database, we observed that a higher enrichment for GT34, CBM25, and GH13 (p < 0.01) the gut microbiota of domestic dogs was related to complex polysaccharide metabolism, reflecting the ability of the gut microbiota of domestic dogs to supply energy from complex dietary polysaccharides through carbohydrate active enzymes (CAZymes) (Cantarel et al. 2009). We also observed that half of the enzymes belong to GTs and five enzymes belong to GHs, while the rest of the enzymes belong to isomaltulose synthase (EC:5.4.99.11) at third level, after classifying the 12 significantly different enzymes obtained from the screening results (Fig. 7). The identified glycosyl transferase enzymes are 4-alpha-glucanotransferase (EC:2.4.1.25) (Chapple 2004; Critchley et al. 2001), glycogen branching enzyme (EC:2.4.1.18) (Abad et al. 2002; Pal et al. 2010), 6^F^-P-sucrose-phosphorylase (EC:2.4.1.329) (Verhaeghe et al. 2014), alpha-1,4-glucan:phosphate-alpha-maltosyltransferase (EC:2.4.99.16) (Elbein et al. 2010; Syson et al. 2011), amylosucrase (EC:2.4.1.4), and cyclomaltodextrin glucanotransferase (EC:2.4.1.19). The identified glycoside hydrolase enzymes are amylo-alpha-1,6-glucosidase (EC:3.2.1.33) (Lee et al. 1970; Nelson et al. 1969), alpha-amylase (EC:3.2.1.1), cyclomaltodextrinase (EC:3.2.1.54), glucodextranase (EC:3.2.1.70), and isoamylase (EC:3.2.1.68) (Gasteiger 2003). It is worth noting that a previous study also confirmed that the *AMY2B* gene, which encodes an alpha-amylase (EC:3.2.1.1), has also been demonstrated to play a key role in increasing the digestibility of starch in domestic dogs (Axelsson et al. 2013). All of these enzymes were related to starch, sucrose, maltose, and glucose metabolism.

According to the results of this study, we determined that most of the bacterial taxa at the family and genus level that have a more significant presence in dogs than in wolves are related to cellulose and starch digestion, and the most significantly different enzymes were associated with carbohydrates, especially amylose, sucrose, and maltose. Therefore, we believe that the significant differences in these bacteria and enzymes have a direct relationship with the changes in the diets of dogs resulting from the domestication of dogs by humans.

In conclusion, we explored the different compositions and functions of the gut microbiota of wolves and domestic dogs using metagenomic sequencing analysis. The findings of our study provided a unique insight into the different functions and compositions of the gut microbiomes between dogs and wolves and increases our understanding of the bacterial ecosystems in canids. However, the sample size in this study was small because of the relatively small number of wolves in China and it is difficult to get proper samples. Further researches are needed to test and verify our findings and in subsequent studies we will continue to enlarge the sample size to obtain more convincing results.

## Abbreviations

GT34: genes encoding glycosyltransferase family 34
CBM25: carbohydrate-binding module family 25
GH13: glycoside hydrolase family 13
Real-time q-PCR: Real-time Quantitative PCR Detecting System
SRA: Sequencing Read Archive
SOAP aligner: Short Oligonucleotide Analysis Package aligner
SOAP denovo: Short Oligonucleotide Analysis Package denovo
ORF: open reading frame
PCA: principal component analysis
NMDS: non-metric multidimensional scaling
KEGG: Kyoto Encyclopedia of Genes and Genomes
CAZy: Carbohydrate-Active Enzymes Database
GHs: glycoside hydrolases
GTs: glycosyl transferases
CBMs: carbohydrate-binding modules
BCAA: branched-chain amino acid
CAZymes: carbohydrate active enzymes

## References

[CR1] Abad MC, Binderup K, Riossteiner J, Arni RK, Preiss J, Geiger JH (2002). The X-ray crystallographic structure of *Escherichia coli* branching enzyme. J Biol Chem.

[CR2] Ames BN (1999). Micronutrient deficiencies. A major cause of DNA damage. Ann N Y Acad Sci.

[CR3] Axelsson E, Ratnakumar A, Arendt ML, Maqbool K, Webster MT, Perloski M, Liberg O, Arnemo JM, Hedhammar Å, Lindblad-Toh K (2013). The genomic signature of dog domestication reveals adaptation to a starch-rich diet. Nature.

[CR4] Bosch G, Hagen-Plantinga EA, Hendriks WH (2015). Dietary nutrient profiles of wild wolves: insights for optimal dog nutrition?. Br J Nutr.

[CR5] Brown DH, Brown BI (1966). [88] Enzymes of glycogen debranching: amylo-1,6-glucosidase (I) and oligo-1,4 → 1,4-glucantransferase (II). Method Enzymol.

[CR6] Bruford MW, Bradley DG, Luikart G (2003). DNA markers reveal the complexity of livestock domestication. Nat Rev Genet.

[CR7] Buchfink B, Xie C, Huson DH (2015). Fast and sensitive protein alignment using DIAMOND. Nat Methods.

[CR8] Cantarel BL, Contiho PM, Rancurel C, Bernard T, Lombard V, Henrissat B (2009). The Carbohydrate-Active EnZymes database (CAZy): an expert resource for glycogenomics. Nucleic Acids Res.

[CR9] Chapple A (2004). A cytosolic glucosyltransferase is required for conversion of starch to sucrose in *Arabidopsis* leaves at night. Plant J.

[CR10] Chen K, Pachter L (2005). Bioinformatics for whole-genome shotgun sequencing of microbial communities. PLoS Comput Biol.

[CR11] Chévezbarrios P, Wiseman AL, Rojas E, Ou CN, Lieberman MW (2000). Cataract development in gamma-glutamyl transpeptidase-deficient mice. Exp Eye Res.

[CR12] Clarke KR (1993). Non-parametric multivariate analyses of changes in community structure. Aust Ecol.

[CR13] Critchley JH, Zeeman SC, Takaha T, Smith AM, Smith SM (2001). A critical role for disproportionating enzyme in starch breakdown is revealed by a knock-out mutation in *Arabidopsis*. Plant J.

[CR14] Diamond J (2002). Evolution, consequences and future of plant and animal domestication. Nature.

[CR15] Eckburg PB, Bik EM, Bernstein CN, Purdom E, Dethlefsen L (2005). Diversity of the human intestinal microbial flora. Science.

[CR16] Elbein AD, Pastuszak I, Tackett AJ, Wilson T, Pan YT (2010). Last step in the conversion of trehalose to glycogen. J Biol Chem.

[CR17] Feng Q, Liang S, Jia H, Stadlmayr A, Tang L, Lan Z, Zhang D, Xia H, Xu X, Jie Z (2015). Gut microbiome development along the colorectal adenoma-carcinoma sequence. Nat Commun.

[CR18] Fu L, Niu B, Zhu Z, Wu S, Li W (2012). CD-HIT: accelerated for clustering the next-generation sequencing data. Bioinformatics.

[CR19] Gao Z, Ma J, Zhang H, Gao Y, Zhao G (1996). Preliminary studies on the food habits of the wolves in eastern Mongolia. Acta Theriol Sin.

[CR20] Gasteiger E (2003). ExPASy: the proteomics server for in-depth protein knowledge and analysis. Nucleic Acids Res.

[CR21] Germonpré M, Sablin MV, Stevens RE, Hedges REM, Hofreiter M, Stiller M, Després VR (2009). Fossil dogs and wolves from Palaeolithic sites in Belgium, the Ukraine and Russia: osteometry, ancient DNA and stable isotopes. J Archaeol Sci.

[CR22] Germonpré M, Lázničková-Galetová M, Sablin MV (2012). Palaeolithic dog skulls at the Gravet-tian Předmostí site, the Czech Republic. J Archaeol Sci.

[CR23] Gharbia SE, Edwards KJ (2015). *Streptobacillus*.

[CR24] Gill SR, Pop M, DeBoy RT, Eckburg PB, Turnbaugh PJ, Samuel BS, Gordon JI, Relman DA, Fraser-Liggett CM, Nelson KE (2006). Metagenomic analysis of the human distal gut microbiome. Science.

[CR25] Giuffra E, Kijas JM, Amarger V, Carlborg O, Jeon JT, Andersson L (2000). The origin of the domestic pig: independent domestication and subsequent introgression. Genetics.

[CR26] Goodman MT, Mcduffie K, Hernandez B, Wilkens LR, Selhub J (2000). Case-control study of plasma folate, homocysteine, vitamin B_12_, and cysteine as markers of cervical dysplasia. Cancer.

[CR27] Greene AC (2014). The family *Desulfuromonadaceae*.

[CR28] Gu S, Fang L, Xu X (2013). Using SOAPaligner for short reads alignment. Curr Protoc Bioinform.

[CR29] Guarner F, Malagelada JR (2003). Gut flora in health and disease. Lancet.

[CR30] Handelsman J, Rondon MR, Brady SF, Clardy J, Goodman RM (1998). Molecular biological access to the chemistry of unknown soil microbes: a new frontier for natural products. Chem Bio.

[CR31] Hehemann JH, Correc G, Barbeyron T, Helbert W, Czjzek M, Michel G (2010). Transfer of carbohydrate-active enzymes from marine bacteria to Japanese gut microbiota. Nature.

[CR32] Huws SA, Kim EJ, Lee MRF, Scott MB, Tweed JKS, Pinloche E, Wallace RJ, Scollan ND (2011). As yet uncultured bacteria phylogenetically classified as *Prevotella*, Lachnospiraceae incertae sedis and unclassified Bacteroidales, Clostridiales and Ruminococcaceae may play a predominant role in ruminal biohydrogenation. Environ Microbiol.

[CR33] Karlsson FH, Tremaroli V, Nookaew I, Bergström G, Behre CJ, Fagerberg B, Nielsen J, Bäckhed F (2013). Gut metagenome in European women with normal, impaired and diabetic glucose control. Nature.

[CR34] Köchl S, Niederstätter H, Parson W (2005). DNA extraction and quantitation of forensic samples using the phenol-chloroform method and real-time PCR. Methods Mol Bio.

[CR35] Kohl KD, Weiss RB, Cox J, Dale C, Denise Dearing M (2014). Gut microbes of mammalian herbivores facilitate intake of plant toxins. Ecol Lett.

[CR36] Larson G, Karlsson EK, Perri A, Webster MT, Ho SYW, Peters J, Stahl PW, Piper PJ, Lingaas F, Fredholm M (2012). Rethinking dog domestication by integrating genetics, archeology, and biogeography. Proc Natl Acad Sci U S A.

[CR37] Lasater PD, Mooers DC (1993) Composition for dog food. US Patent 5,200,218, 6 Apr 1993

[CR38] Lee EYC, Carter JH, Nielsen LD, Fischer EH (1970). Purification and properties of yeast amylo-1,6-glucosidase-oligo-1,4.far. 1,4-glucantransferase. Biochemistry.

[CR39] Leisner JJ, Laursen BG, Prévost H, Drider D, Dalgaard P (2007). *Carnobacterium*: positive and negative effects in the environment and in foods. Fems Microbiol Rev.

[CR40] Li J, Jia H, Cai X, Zhong H, Feng Q, Sunagawa S, Arumugam M, Kultima JR, Prifti E, Nielsen T (2014). An integrated catalog of reference genes in the human gut microbiome. Nat Biotechnol.

[CR41] Lopezsiles M, Khan TM, Duncan SH, Harmsen HJ, Garciagil LJ, Flint HJ (2012). Cultured representatives of two major phylogroups of human colonic *Faecalibacterium prausnitzii* can utilize pectin, uronic acids, and host-derived substrates for growth. Appl Environ Microbiol.

[CR42] Luo R, Liu B, Xie Y, Li Z, Huang W, Yuan J, He G, Chen Y, Qi P, Liu Y (2012). SOAPdenovo2: an empirically improved memory-efficient short-read de novo assembler. Gigascience.

[CR43] Mende DR, Waller AS, Sunagawa S, Järvelin AI, Chan MM, Arumugam M, Raes J, Bork P (2012). Assessment of metagenomic assembly using simulated next generation sequencing data. PLoS ONE.

[CR44] Nelson TE, Kolb E, Larner J (1969). Purification and properties of rabbit muscle amylo-1,6-glu-cosidase-oligo-1,4-1,4-transferase. Biochemistry.

[CR45] Novelli ELB, Santos PP, Assalin HB, Souza G, Rocha K, Ebaid GX, Seiva FRF, Mani F, Fernandes AA (2009). *N*-acetylcysteine in high-sucrose diet-induced obesity: energy expenditure and metabolic shifting for cardiac health. Pharmacol Res.

[CR46] Oh J, Byrd AL, Deming C, Conlan S, Kong HH, Segre JA (2014). Biogeography and individuality shape function in the human skin metagenome. Nature.

[CR47] Ovodov ND, Crockford SJ, Kuzmin YV, Higham TFG, Hodgins GWL, Plicht JVD (2011). A 33,000-year-old incipient dog from the Altai Mountains of Siberia: evidence of the earliest domestication disrupted by the Last Glacial Maximum. PLoS ONE.

[CR48] Pal K, Kumar S, Sharma S, Garg SK, Alam MS, Xu HE, Agrawal P, Swaminathan K (2010). Crystal Structure of full-length *Mycobacterium tuberculosis* H37Rv glycogen branching enzyme: insights of N-terminal beta-sandwich in substrate specificity and enzymatic activity. J Biol Chem.

[CR49] Panaro NJ, Yuen PK, Sakazume T, Fortina P, Kricka LJ, Wilding P (2000). Evaluation of DNA fragment sizing and quantification by the agilent 2100 bioanalyzer. Clin Chem.

[CR50] Perri A (2016). A wolf in dog’s clothing: initial dog domestication and Pleistocene wolf variation. J Archaeol Sci.

[CR51] Rampelli S, Schnorr SL, Consolandi C, Turroni S, Severgnini M, Peano C, Brigidi P, Crittenden AN, Henry AG, Candela M (2015). Metagenome sequencing of the Hadza hunter-gatherer gut microbiota. Curr Biol.

[CR52] Rowe JB, Choct M, Brown W, Day K (1997) Variation in the carbohydrate composition of dog food. http://www.livestocklibrary.com.au/bitstream/handle/1234/19804/97_242.pdf?sequence=1

[CR53] Rubin CJ, Zody MC, Eriksson J, Meadows JR, Sherwood E, Webster MT, Jiang L, Ingman M, Sharpe T, Ka S (2010). Whole-genome resequencing reveals loci under selection during chicken domestication. Nature.

[CR54] Savolainen P, Zhang Y, Luo J, Lundeberg J, Leitner T (2002). Genetic evidence for an East Asian origin of domestic dogs. Science.

[CR55] Schnorr SL, Candela M, Rampelli S, Centanni M, Consolandi C, Basaglia G, Turroni S, Biagi E, Peano C, Severgnini M (2014). Gut microbiome of the Hadza hunter-gatherers. Nat Commun.

[CR56] Silva NPD, Souza FISD, Pendezza AI, Fonseca FLA, Hix S, Oliveira AC, Sarni ROS, D’Almeida V (2013). Homocysteine and cysteine levels in prepubertal children: association with waist circumference and lipid profile. Nutrition.

[CR57] Smith C, Osborn A (2009). Advantages and limitations of quantitative PCR (Q-PCR)-based approaches in microbial ecology. FEMS Microbiol Ecol.

[CR58] Syson K, Stevenson CE, Rejzek M, Fairhurst SA, Nair A, Bruton CJ, Field RA, Chater KF, Lawson DM, Bornemann S (2011). Structure of *Streptomyces* maltosyltransferase GlgE, a homolo-gue of a genetically validated anti-tuberculosis target. J Biol Chem.

[CR59] Thalmann O, Shapiro B, Cui P, Schuenemann VJ, Sawyer SK, Grennfield DL, Germonpré MB, Sablin MV, López-Giráldez F, Domingo-Roura X (2013). Complete mitochondrial genomes of ancient canids suggest a European origin of domestic dogs. Science.

[CR60] Verhaeghe T, Aerts D, Diricks M, Soetaert W, Desmet T (2014). The quest for a thermostable sucrose phosphorylase reveals sucrose 6′-phosphate phosphorylase as a novel specificity. Appl Microbiol Biotechnol.

[CR61] Wang GD, Zhai W, Yang HC, Fan RX, Cao X, Zhong L, Wang L, Liu F, Wu H, Cheng LG (2013). The genomics of selection in dogs and the parallel evolution between dogs and humans. Nat Commun.

[CR62] Werner L, Kai F (2015). *Desulfuromusa*.

[CR63] White JR, Nagarajan N, Pop M (2009). Statistical methods for detecting differentially abundant features in clinical metagenomic samples. PLoS Comput Biol.

[CR64] Wu X, Zhang H, Chen J, Shang S, Yan J, Chen Y, Tang X, Zhang H (2017). Analysis and comparison of the wolf microbiome under different environmental factors using three different data of Next Generation Sequencing. Sci Rep.

[CR65] Yan W, Zhang H, Yang H, Dou H, Shen X (2006). Seasonal diet of wolves in the Dalaihu natural reserve, Inner Mongolia. Chin J Zool.

[CR66] Zaunmüller T, Eichert M, Richter H, Unden G (2006). Variations in the energy metabolism of biotechnologically relevant heterofermentative lactic acid bacteria during growth on sugars and organic acids. Appl Microbiol Biotechnol.

[CR67] Zeder MA (2015). The domestication of animals. J Anthropol Res.

[CR68] Zhu L, Wu Q, Dai J, Zhang S, Wei F (2011). Evidence of cellulose metabolism by the giant panda gut microbiome. Proc Natl Acad Sci U S A.

[CR69] Zohary D, Hopf M (2000). Domestication of plants in the Old World. J Appl Ecol.

