# Correction to: Changes in feeding habits promoted the differentiation of the composition and function of gut microbiotas between domestic dogs (*Canis lupus familiaris*) and gray wolves (*Canis lupus*)

**DOI:** 10.1186/s13568-021-01239-z

**Published:** 2021-06-03

**Authors:** Tianshu Lyu, Guangshuai Liu, Huanxin Zhang, Lidong Wang, Shengyang Zhou, Huashan Dou, Bo Pang, Weilai Sha, Honghai Zhang

**Affiliations:** 1 Qufu Normal University, Qufu, 273165 China; 2 Ocean University of China, Tsingtao, 266100 China; 3 Dalai Lake National Nature Reserve Management Bureau, Hailar, 021000 China

## Correction to: AMB Expr 8:123 (2018) https://doi.org/10.1186/s13568-018–0652-x

Following publication of the original article (Lyu et al. 2018), the authors would like to correct the accession number in the last sentence of the "Metagenomic sequencing" section of the article.

The correct number is SRP149020 instead of SRP179020. This has been corrected with this erratum.
